# Neuromodulatory control of energy reserves in dopaminergic neurons

**DOI:** 10.1073/pnas.2523019122

**Published:** 2025-12-05

**Authors:** Camila Pulido, Matthew S. Gentry, Timothy A. Ryan

**Affiliations:** ^a^Department of Biochemistry and Biophysics, Weill Cornell Medicine, New York, NY 10165; ^b^Aligning Science Across Parkinson’s Collaborative Research Network, Chevy Chase, MD 20815; ^c^Department of Biochemistry and Molecular Biology, University of Florida, Gainesville, FL 32610-0208

**Keywords:** synapse, glycogen, dopamine, ATP

## Abstract

The brain is a metabolically vulnerable organ as neurons have both high resting metabolic rates and the need for local rapid conversion of carbon sources to ATP during activity. Midbrain dopamine neurons are thought to be particularly vulnerable to metabolic perturbations, as a subset of these are the first to undergo degeneration in Parkinson’s disease, a neurodegenerative disorder long suspected to be in part driven by deficits in mid-brain bioenergetics. In skeletal muscle, energy homeostasis under varying demands is achieved in part by its ability to rely on glycogen as a fuel store, whose conversion to ATP is under hormonal regulatory control. In neurons, however, the absence of easily observable glycogen granules has cast doubt on whether this fuel store is operational, even though brain neurons express the key regulatory enzymes associated with building or burning glycogen. We show here that in primary mid-brain dopaminergic neurons, glycogen availability is under the control of dopamine autoreceptors, such that dopamine itself provides a signal to store glycogen. We find that when glycogen stores are present, they provide remarkable resilience to dopamine nerve terminal function under extreme hypometabolic conditions, but loss of this dopamine-derived signal, or impairment of access to glycogen, makes them hypersensitive to fuel deprivation. These data show that neurons can use an extracellular cue to regulate local metabolism and suggest that loss of dopamine secretion might make dopamine neurons particularly subject to neurodegeneration driven by metabolic stress.

## Significance

This work demonstrates that neurons have an active glycogen metabolism that can support synapse function during metabolic stress. Furthermore, we found that access to glycogen is actively shaped by local neuromodulation. This provides a potential explanation for why different neuronal subtypes exhibit unequal vulnerability to metabolic stressors and why in Parkinson’s Disease dopamine neurons might degenerate faster as loss of dopamine signaling in turn would deprive them of a backup fuel.

Glycogen, an osmotically neutral polymer of glucose, is the major glucose reserve in eukaryotes, and defects in glycogen metabolism and structure lead to disease. The fundamental understanding of glycogen metabolism stems from pioneering studies in liver and muscle over the last 80 y (1). In liver, glycogen serves as the fuel store to maintain blood glucose homeostasis, where insulin and glucagon mediate the balance of glycogen synthesis or breakdown resulting in net glucose uptake or export from this organ. Muscle uses glycogen as a fuel store that can rapidly mobilize phosphorylated glucose from the glucose polymer under the control of epinephrine (2). The liberated glucose is then oxidized to ATP and pyruvate to meet the bioenergetic needs of muscle contraction. Neurons, like muscle, have bioenergetic needs that vary over time and must synthesize ATP on demand to meet the needs of the neuronal molecular machinery engaged by electrical activity (3). Failure to meet the local ATP needs in axons during activity leads to rapid collapse of synaptic function (4, 5). Paradoxically, the brain, and in particular neurons, have long been considered to rely minimally on glycogen stores despite expressing the key enzymes associated with glycogen metabolism (6). Part of this view stems from the fact that the classic way to visualize glycogen is by electron microscopy. In resting muscle, for example, glycogen polymers of up to 50,000 glucose molecules are synthesized and can be easily recognized as a classic granule signature (7), but smaller granules are not easily distinguished from other electron-dense subcellular material. Although glycogen granules are occasionally visualized in astrocytes, particularly under certain forms of anesthesia (8), they are not readily observed in neurons, leading to the prevailing view that most glycogen metabolism in the brain resides exclusively in nonneuronal cells. It is important to note, however, that the abundance of glycogen reflects the steady-state balance of synthesis and breakdown, and low steady-state abundance may simply reflect a constant flux through the pathway. The consequence of disrupting glycogen metabolism in the brain on neuronal function has usually been interpreted as evidence for the shuttling of a glycolytic end product, lactate from astrocytes (9). Recent quantitative evidence, however, does not support the idea that astrocytes, as a rule, provide lactate during or after electrical activity to support neuronal function (10, 11). Additionally, neuron-specific genetic ablation of critical proteins needed for glycogen metabolism leads to significant impairment in cognitive function (12), supporting the notion that neuronal glycogen metabolism plays an important role in normal brain function. Although glycogen metabolism would be expected to lead to increased ATP production, as is likely in muscle treated with epinephrine, recent evidence indicates that under some circumstances, glycogen metabolism is shunted to the pentose phosphate pathway, generating NADPH required for glutathione activity, in turn providing protection against oxidative stress (13, 14). To help clarify whether or not neuronal glycogen metabolism plays a role similar to that in muscle, we made use of primary neurons, where it is possible to acutely drive bioenergetic needs with electrical activity while monitoring intracellular ATP dynamics (15) or synaptic function (16). Here, we show that primary rat mid-brain dopamine neurons can rely on glycogen to sustain nerve terminal function in the absence of glucose, but that the ability to do so depends on the previous activity of dopamine autoreceptors (D2R). Pretreatment of these neurons with a D2R antagonist leads to depletion of glycogen stores and makes their nerve terminals hypersusceptible to loss of extracellular glucose, and unable to sustain cytoplasmic ATP production. These data provide direct evidence that glycogen, even in the absence of external glucose, can meet the bioenergetic needs of synapses under prolonged stimulation, but access to glycogen in turn is under neuromodulatory control. The data suggest that as dopamine secretion becomes impaired in the brain, the loss of the autocrine signal in turn will make dopamine neurons more susceptible to metabolic stressors. The critical importance of D2Rs in regulating this energy reserve additionally suggests that this may underlie the susceptibility of presymptomatic patients to drug-induced Parkinsonism driven by atypical antipsychotics that target D2Rs (17, 18).

## Results

### Primary Dopaminergic Axon Terminals are Highly Resistant to Glucose Deprivation.

**Fig. 1.**
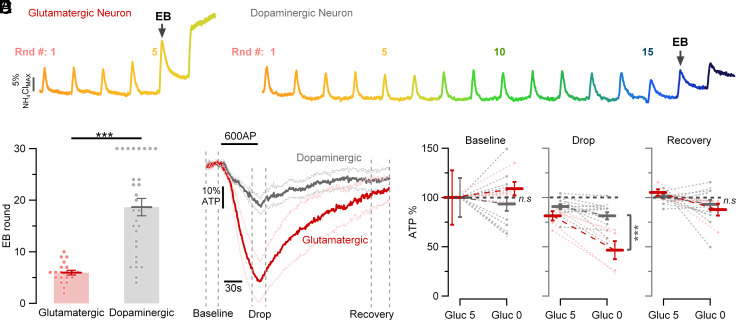
Dopaminergic axonal terminals are more resilient to fuel deprivation compared to glutamatergic terminals. (*A* and *B*) Synaptic function measured in neurons expressing Syphy-pH in the absence of glucose (0 mM glucose). Color-coded traces represent responses to one stimulus round (50 Aps, 10 Hz) applied every minute (from orange = first round to dark blue = 17th round). Responses are normalized to sensor expression using ammonium chloride measurements. (*A*) Syphy-pH trace of six stimulus rounds in a glutamatergic neuron. (*B*) Syphy-pH trace of seventeen stimulus rounds in a dopaminergic neuron. (*C*) Number of rounds of stimulation before EB exceeds 50% is smaller in glutamatergic neurons (red dots, *n* = 20) than dopaminergic neurons (gray dots, *n* = 29): mean ± SEM: 5.95 ± 0.45 rounds vs. 18.66 ± 1.69 rounds, where 8 out 29 dopaminergic neurons never arrested for all 30 rounds of stimulation. (*D*) Average axonal ATP changes to 600 APs, 10 Hz stimulation in neurons expressing iATPSnFR2.0 in the absence of glucose (0 mM glucose), normalized to prestimulation baseline. Dopaminergic (gray trace, *n* = 14) vs. glutamatergic axonal terminals (red trace, *n* = 5). (*E*) ATP dynamics before (Baseline), during (Drop), and after (Recovery) electrical activity in 5 mM glucose (Gluc 5) and after 5 min incubation without glucose (Gluc 0). Dopaminergic axons generate sufficient ATP during and after electrical activity to sustain greater SV recycling than glutamatergic axons. ATP drop during stimulation in the absence of glucose in dopaminergic vs glutamatergic: mean ± SEM: 18.64 ± 3.69 vs. 53.4 ± 9.16, respectively. ***P < 0.001, nonsignificant as ns, Wilcoxon–Mann–Whitney test.

As mid-brain dopaminergic neurons are known to undergo preferential degeneration in Parkinson’s disease (PD), and PD is thought in part to be driven by deficits in brain bioenergetics, we sought to examine the vulnerability of dopamine neurons to specific metabolic stresses using a reductionist approach in vitro. We previously developed a quantitative optical approach to examine the sensitivity of synaptic performance to metabolic perturbations, using the dependence of vesicle recycling on ATP availability (16, 19). Here, we made use of the tyrosine hydroxylase (TH) promoter (20) to specifically enrich for expression of different genetically encoded functional indicators in dissociated mid-brain rat dopamine neurons (*SI Appendix*, Fig. S1) to examine their sensitivity to metabolic stress, in particular to hypoglycemic conditions. We examined the kinetics of synaptic vesicle (SV) recycling during repeated bouts of stimulation (50 APs, 10 Hz) delivered at minute intervals in the absence of external glucose. Consistent with our previously work (19), this protocol leads to an arrest in SV retrieval in hippocampal neurons after ~5 rounds of stimulation (Fig. 1*A*). To characterize the variation in synaptic endurance across cells, we measured the fraction of the exocytic signal that remained after a defined post stimulus time period (3 × the decay time constant in one round of stimulation in 5 mM glucose, 3τ), which we term the percentage of endocytic block (EB) (=~5% in the first round by definition for a perfect exponential decay). For a given neuron, we determined the number of stimulus rounds it took for SV recycling to fail to retrieve 50% of the exocytic signal at this time period (EB in the trace), which in hippocampal neurons was, on average, 5.95 rounds ± 0.45 (*n* = 20; Fig. 1*C*), consistent with previous findings (19). In contrast, we were surprised to find that dopaminergic nerve terminals exhibited a much greater resilience to the withdrawal of glucose and sustained robust SV recycling on for many rounds of repeated burst of AP firing, where a large subset (8/29) failed to arrest for all 30 rounds of stimulation (Fig. 1 *B* and *C*).

The metabolic resilience of primary dopamine neurons strongly implies that even in the absence of external glucose, their nerve terminals generate sufficient ATP during and after electrical activity to sustain SV recycling. To test this hypothesis, we expressed a recently developed genetically encoded fluorescent ATP sensor, iATPSnFR2.0 (15) in dopamine neurons. As shown previously, when glutamatergic hippocampal neurons are subjected to prolonged stimulation in the absence of external glucose, ATP levels drop precipitously (~53%) and recover only slowly after the cessation of activity, consistent with the idea that there is no readily available oxidizable carbon source to synthesize ATP as it is consumed (Fig. 1 *D* and *E*). In contrast, in dopamine neurons, ATP levels decrease by only ~18% using the same stimulus paradigm and readily returned to baseline following electrical activity (Fig. 1 *D* and *E*). These data thus demonstrate that primary mid-brain dopamine neurons deploy a mechanism that allows them to generate ATP during activity in the complete absence of an explicit external carbon source that is sufficient to sustain robust nerve terminal function.

### Dopamine Neurons and their Nerve Terminals Show Robust Expression of Glycogen Synthase and Glycogen.

**Fig. 2.**
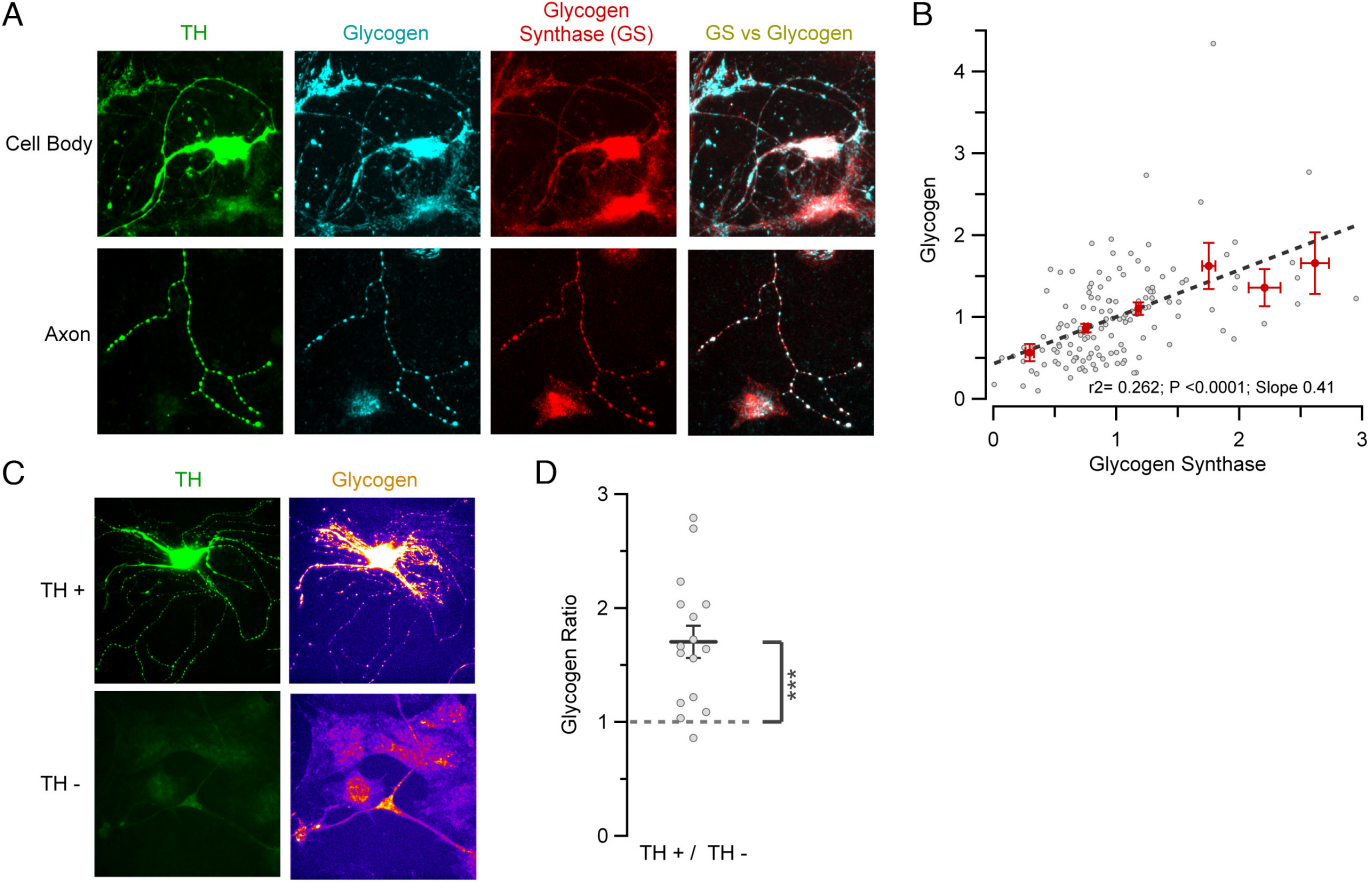
Glycogen synthase is expressed in dopaminergic axon terminals, enabling local glycogen storage. (*A*) Representative cell body (*Top*) and axon (*Bottom*) images tripe-labeled with anti-TH (green), anti-Glycogen (cyan), and anti-Glycogen Synthase (GS; red) shows that dopaminergic terminals have the enzymatic machinery to synthesize and store glycogen. Overlay image between GS vs. glycogen is shown in the right column. (*B*) Normalized glycogen storage levels of cell bodies from a mix of TH-positive and -negative populations (gray dots; *n* = 138 cell bodies from five cultures) are linearly correlated (black dashed line) with the normalized GS expression levels. Binned average by a 0.5 window (red dots; Mean ± SEM). (*C*) Representative images of a dopaminergic (TH positive) vs. a nondopaminergic cell body (TH negative; green) from a same coverslip and their respective glycogen level content (color-code from dark violet = low levels to yellow = high levels; same scale top and bottom). (*D*) On average, dopaminergic neurons store more glycogen than nondopaminergic populations. TH+/TH− ratio per dish: mean ± SEM: 1.7 ± 0.142; *n* = 16 coverslips from nine cultures.

The remarkable metabolic resilience of dopamine neurons even when bathed in glucose-free saline suggests that these neurons have access to an intracellular source of combustible carbon to maintain synaptic function. We tested the idea that glycogen might be the relevant carbon source. Although electron microscopy is the traditional approach to detect the classic granule signature, we reasoned that this might be difficult to implement for in vitro studies, as primary dissociated dopamine neurons are part of a mixed cell type, that include both astrocytes and non-TH expressing neurons. Additionally, when glycogen granules are only a few nanometers in size they are indistinguishable from other electron-dense material in the cytoplasm. To overcome this problem, we utilized a monoclonal antibody previously shown to have very high affinity for both purified glycogen granules as well as enzymatically digested glycogen (21, 22). We triple-labeled primary mid-brain dissociated neuron cultures with the anti-Glycogen, anti-TH, and an antibody for Glycogen Synthase (GS). These experiments showed directly that dopamine neurons (TH-positive) have robust expression of GS and have an accumulation of glycogen (Fig. 2*A*). Glycogen and GS were distributed through the somato-dendritic regions and in axons, including at presynaptic varicosities. Both the amount of glycogen and GS detectable in individual cell somas from a mix population (TH positive and negative) was quite variable with more than an order of magnitude difference in each of these across cells (Fig. 2*B*). Analysis of the paired normalized intensities of glycogen and GS over >130 individual cells showed that there is a linear correlation between accumulation of glycogen and the expression of GS (Fig. 2*B*). We hypothesized that dopamine neurons were particularly enriched in glycogen. To examine this, we compared the intensities of glycogen staining on the same coverslip on cell bodies that were TH positive and negative (Fig. 2*C*). This analysis showed that on average dopamine neurons have ~ 70% more glycogen expression than non-TH containing cells (Fig. 2*D*).

### Primary Dopamine Neurons Utilize Their Own Stored Glycogen to Sustain Function Under Hypoglycemic Conditions.

**Fig. 3.**
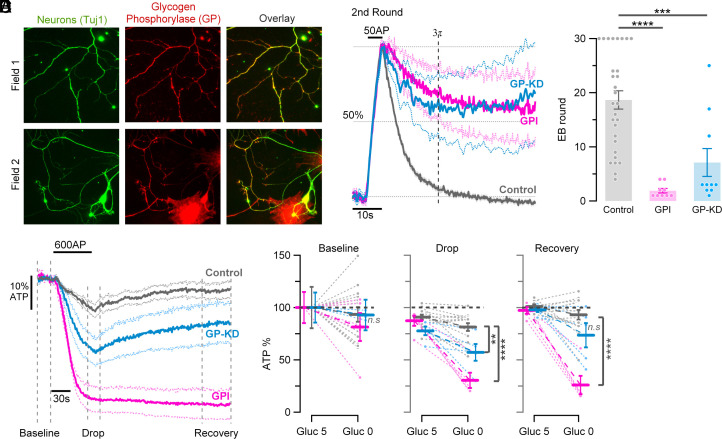
Dopaminergic terminals locally degrade glycogen into fuel to sustain synaptic function under hypoglycemic conditions. (*A*) Representative images of axons (Field 1 and 2) and somato-dendric regions (Field 2) labeled with anti-Tuj1 (green) and anti-Glycogen Phosphorylase (GP; red) show that axonal terminals locally express the enzymatic machinery capable of breaking down glycogen into a fuel source. Overlay images are shown on the right column. (*B*) Average traces in the absence of glucose at the second round of stimulation (50 AP, 10 Hz) in control neurons (gray trace; *n* = 29), neurons treated with GPI (magenta trace; *n* = 9) and neurons suppressing expression of GP (GP-KD; blue trace; *n* = 10). Responses are normalized to the peak. Vertical dashed line represents three times *T* measured at 5 mM glucose response. Horizontal dashed line represents 50% retrieval of the exocytic signal. (*C*) Number of rounds of stimulation before EB exceeds 50% is smaller in neurons with GP function impairment: GPI treatment and GP-KD (magenta and blue dots, respectively), than in neurons expressing functional GP (gray dots): mean ± SEM: 1.89 ± 0.42 rounds vs. 7.1 ± 2.58 rounds vs. 18.66 ± 1.69 rounds, respectively, where 8 out 29 dopaminergic neurons never arrested for all 30 rounds of stimulation. (*D*) Average axonal ATP changes to 600 APs, 10 Hz stimulation in neurons expressing iATPSnFR2.0 in the absence of glucose, normalized to prestimulation baseline. Control (gray trace, *n* = 14) vs. GPI treatment (magenta, *n* = 5) and GP-KD (blue, *n* = 5) neurons shows that dopaminergic axons depend on GP function to breakdown glycogen to be able to generate sufficient ATP to sustain activity. (*E*) Comparison of ATP percentage before (Baseline), during (Drop), and after (Recovery) electrical activity in 5 mM glucose (Gluc 5), and after 5 min in a glucose free saline solution (Gluc 0). ****P< 0.0001, ***P< 0.001, **P< 0.01, nonsignificant as ns, Wilcoxon–Mann–Whitney test.

The balance of glycogen abundance in tissues is set in part by the activities of GS and glycogen phosphorylase (GP), with GP being required to liberate phosphorylated glucose monomers from the glycogen granule. GP activity is controlled by phosphorylase kinase whose activity is allosterically controlled by a combination of Ca^2+^ ions and protein kinase A (23). However, the detailed mechanism that engage the control of glycogen metabolism on short timescales remain poorly understood. Immunostaining for GP shows that, like GS, it is expressed throughout axons and somato-dendric regions of primary mid-brain neurons (Fig. 3*A*). In order to test whether glycogenolysis is supporting SV recycling and ATP production we made use of both an shRNA that led to a 50% reduction of GP (*SI Appendix*, Fig. S3) as well as a small molecule glycogen phosphorylase inhibitor (GPI) and examined their impact on SV recycling and ATP production in the absence of glucose during electrical activity. Unlike in untreated control dopamine neurons, acute application of GPI or loss of GP expression (GP KD), resulted in SV recycling in the absence of glucose to arrest within two (GPI) or seven (GP KD) rounds of stimulation (Fig. 3 *B* and *C*). Consistent with these observations, measurements of cytosolic ATP using iATPSnFR2.0 showed prolonged electrical activity in dopamine neurons treated with GPI led to a ~70% reduction in ATP that failed to recover, while stimulation in GP KD neurons ATP levels dropped by ~ 40% and recovered only very slowly following stimulation (Fig. 3 *D* and *E*).

### Glycogen Storage in Dopamine Neurons Is Controlled by D2 Autoreceptors.

**Fig. 4.**
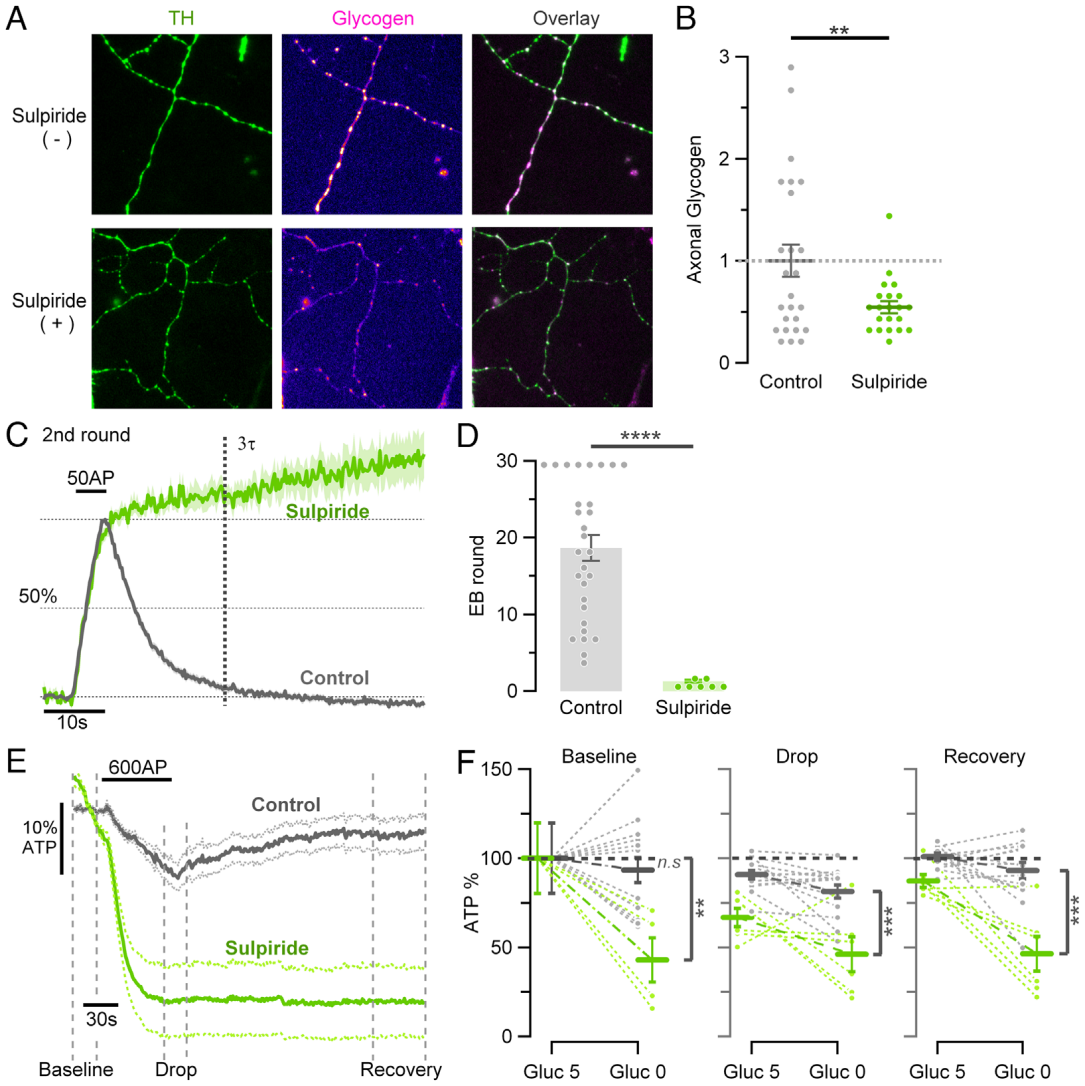
Local glycogen storage levels in dopamine neurons are modulated by D2 autoreceptors. (*A*) Representative images from dopamine axon terminals (anti-TH; green) with (*Bottom*) or without (*Top*) overnight 1 μM sulpiride treatment; and their respective glycogen level content (color-code from dark violet = low levels to yellow = high levels; same scale top and bottom). (*B*) Average glycogen expression from axonal terminals normalized to control (gray dots; *n* = 25 neurons from three cultures), shows that glycogen storage levels decrease by a 45.4% ± 5.9 when neurons were treated with sulpiride (green dots; *n* = 21 from same cultures than control). (*C*) Average traces at the second round of stimulation (50 AP, 10 Hz) in the absence of glucose in control neurons (gray trace; *n* = 29) vs. neurons treated with sulpiride (green trace; *n* = 7). Responses are normalized to the peak. Vertical dashed line represents three times *t* measured at 5 mM glucose response. Horizontal dashed line represents 50% retrieval of the exocytic signal. (*D*) Number of rounds of stimulation before EB exceeds 50% is smaller in sulpiride-treated neurons (green dots) than control neurons (gray dots): mean ± SEM: 1.3 ± 0.18 rounds vs. 18.66 ± 1.69 rounds. Where 8 out 29 dopaminergic neurons never arrested for all 30 rounds of stimulation. (*E*) Average axonal ATP changes to 600 APs, 10 Hz stimulation in neurons expressing iATPSnFR2.0 in the absence of glucose, normalized to prestimulation baseline. Control (gray trace, *n* = 14) vs. sulpiride-treated neurons (green, *n* = 6) shows that D2-autoreceptor signaling controls glycogen storage, and that sulpiride reduces this storage to levels insufficient for sustaining SV function during and after activity. (*F*) Comparison of ATP percentage before (Baseline), during (Drop), and after (Recovery) electrical activity in 5 mM glucose (Gluc 5) and after 5 min in a glucose-free saline solution (Gluc 0). ****P< 0.0001, ***P< 0.001, **P< 0.01, nonsignificant as ns, Wilcoxon–Mann–Whitney test.

In muscle, the balance of glycogenesis vs. glycogenolysis is controlled by the concentration of cAMP and Ca^2+^ ions as together they lead to the activation of GP. We reasoned that GPCRs that are coupled to Gi to inhibit cAMP production could potentially tilt the balance of activity toward glycogen storage. Mid-brain dopamine neurons express D2 receptors (24), a variant of dopamine receptor coupled to Gi/o (25). As dissociated primary dopamine neurons are likely exposed to a constant level of dopamine autocrine stimulation, perhaps similar to what occurs during tonic firing in the striatum (26), we reasoned that this stimulation of D2Rs might be sufficient to drive glycogen storage and form the basis of the metabolic resilience. To test this hypothesis, we made use of sulpiride, a clinically used antipsychotic D2R antagonist. Consistent with this idea, incubating primary dopamine neurons overnight in 1 μM sulpiride led to a 50% reduction in glycogen content in axonal varicosities (Fig. 4 *A* and *B*) and a ~30% reduction in cell bodies (*SI Appendix*, Fig. S4 *A* and *B*). Notably, sulpiride had no impact on glycogen levels in nondopaminergic neurons (*SI Appendix*, Fig. S4*B*). To determine whether this reduction in glycogen levels impacted axonal bioenergetics, we used our synaptic endurance test (Fig. 1 *A* and *B*) to examine the sensitivity of SV recycling to removal of glucose. These experiments showed that unlike with control neurons, previous treatment with sulpiride and the loss of glycogen, rendered primary dopamine neurons incapable of sustaining SV recycling for more than two rounds of stimulation (Fig. 4 *C* and *D*). Measurements of intracellular ATP revealed that neurons treated with sulpiride, even prior to stimulation, could not sustain ATP levels upon switching to a glucose-free saline solution. During AP firing, the decline in ATP continued at an even faster rate and failed to recover in the poststimulus period in dopamine neurons treated with sulpiride (Fig. 4 *C*, *E*, and *F*). These data strongly suggest that dopamine neurons, in the absence of access to glycogen, now are much more metabolically vulnerable. By comparison, hippocampal neurons do not change their metabolic sensitivity upon block with GP (*SI Appendix*, Fig. S2).

## Discussion

The basis for selective vulnerability of certain cell types in the brain remains a central question for understanding the molecular origins of neurodegeneration. This question is particularly relevant in PD, as the degree of degeneration of dopamine neurons in the Substantia Nigra pars compacta (SNc) are strongly correlated with duration of the disease (27, 28). Several important clues point to the possibility that PD, and the selective vulnerability of dopamine neurons may arise from bioenergetic deficits. Quantitative analysis of glucose metabolism suggests that brain hypoglycemia may be an early predictor of PD (29, 30), and genetic studies in humans in the last three decades has successfully identified over 20 monogenic drivers of familial PD including many that are implicated either directly or indirectly in impacting cellular bioenergetics. Several of the disease-driving genetic variants participate in mitochondrial quality control (31), e.g., Parkin (*PARK2*), and Pink1 (*PARK6*) (31) or are themselves bona fide mitochondrial proteins, e.g., CHCHCD2 (*PARK22*) (32). Furthermore, recent evidence has accumulated that several other genetic drivers of PD either impair neuronal glycolysis, e.g., DJ-1 (*PARK7*) and PGK1 (*PARK12*) or create a metabolic burden in axons (16). An FDA-approved treatment of benign prostate hyperplasia, terazosin, was discovered to have an off-target enhancement of glycolysis (33). Terazosin in turn has proven remarkably effective in several animal models of PD (34) and retrospective epidemiology has shown that terazosin significantly reduces the risk of developing PD in humans compared to different BPH treatments with the same intended molecular target (35). These findings strongly support the idea that both monogenic and idiopathic PD have a strong bioenergetic component, in turn pointing to the need to examine the bioenergetic properties of mid-brain dopamine neurons. The data we show here demonstrate that in vitro, contrary to expectations, nerve terminal function in primary dopamine neurons is much more resilient to fuel withdrawal than primary hippocampal neurons. Although it is speculated that individual dopaminergic nerve terminals release fewer total vesicles than nerve terminals of excitatory neurons (36) that in turn could lead to lower energetic demands associated with vesicle recycling and endocytosis, we traced the origin of this resilience to the fact that primary dopamine neurons under these conditions were reliant on stored glucose in the form of glycogen, but that the availability of such a store was itself dependent on whether the neurons had previously activated their D2Rs. Previous work has shown that glycogen was protective against hypoxia in neurons (6) indicating that when available, glycogen would be protective. Remarkably, this resilience to fuel withdrawal comes at a price, as if either the glycogen stores are depleted (Fig. 4*D*) or access to them is blocked (*SI Appendix*, Fig. S2*C*), primary dopamine neurons are more sensitive to fuel restriction. These data lead us to propose that the combination of a reliance on glycogen coupled with the autocrine nature of glycogen control may explain the general susceptibility of dopamine neurons in SNc to metabolic stressors, as once these neurons stop secreting sufficient dopamine such that resting levels in the striatum become depleted, this in turn leads to a depletion of glycogen stores resulting effectively to an positive feedback that leads to even greater metabolic stress in dopamine neurons, exacerbated nerve terminal function, and further dopamine depletion. Consistent with this idea, human disorders involving glycogen storage impairment have been associated with parkinsonian features. For example, an adult-onset Lafora disease patient was reported to exhibit bradykinesia, rigidity, and dopaminergic deficits based on DAT imaging (37), while a teenage patient carrying an *NHLRC1* mutation presented with early parkinsonian symptoms (38).

In muscle, it has long been appreciated that glycogen stores serve as an important component of the “fight or flight” response, whereby adrenaline secretion drives glycogenolysis in turn boosting the bioenergetic capacity of the muscle. Our work demonstrates that neurons appear to also make use of neuromodulation to control their bioenergetic capacity by providing a signal to build energy reserves. At present, we do not know whether neurons, and in particular dopamine neurons, utilize neuromodulation (presumably by elevating cAMP) to facilitate glycogenolysis or if they simply rely on an intrinsic Ca^2+^modulation (perhaps via a Ca^2+^-sensitive adenylate cyclase) of this activation. Given the widespread expression of GPCRs in the brain, it is tempting to speculate that part of this may be used to locally gate both storage (via Gi activation) and use of energy reserves (via Gs activation), that in turn will help control cognitive function. An intriguing possibility for future investigation is that D1 receptor–mediated Gs activation could acutely engage the cAMP–PKA pathway to trigger glycogen breakdown during periods of high metabolic demand.

We do not understand at this point the molecular basis for the hypersensitivity of dopamine neurons to metabolic stress when deprived access to glycogen. It may reflect a fundamental difference in some aspect of the molecular organization the glycolytic machinery that is well adapted to direct delivery of phosphorylated glucose from the glycogen store but poorly adapted to extracellular glucose delivery. Further studies to understand this point are warranted as well as the general applicability of these findings to other neuron types both in vitro and in vivo. Our work is consistent with the observation that specific loss of glycogen machinery in neurons in vivo degrades neuronal function in rodents and worms (39, 40) suggesting that glycogen use by neurons is a fundamental property and means of regulating bioenergetic capacity in the brain.

## Materials and Methods

All protocols for this study have been deposited at https://doi.org/10.17504/protocols.io.ewov11q42vr2/v1.

### Reagents.

Chemical reagents were purchased from MilliporeSigma. GPI was purchased from Cayman Chamical (18578). Sulpiride was from MilliporeSigma (S7771). Antibodies used are anti-TH in rabbit (Millipore, 657012, RRID: AB_2201407, 1:1,000), anti-TH in sheep (Millipore, AB1542, RRID: AB_90755, 1:1,000), a-tubulin-*β*III (R&D Systems, MAB1195, RRID: AB_357520, 1:1,000), anti-Glycogen Phosphorylase (Synaptic Systems, 255 003, RRID:AB_2619966, 1:500), anti-Glycogen Synthase 1 (Proteintech, 10566-1-AP, RRID: AB_2116401, 1:500), IV58B6 anti-Glycogen (provided by Dr. M. Gentry, 1:500), and anti-TagFP nanobody conjugated with ATTO488 (NanoTag Biotechnologies, N0502-At488-L, RRID: AB_2744623; 1:500). Alexa Fluor–conjugated fluorescent secondary antibodies were obtained from Life Technologies and used at 1:500.

### Animals.

All experiments involving animals were performed in accordance with protocols approved by the Weill Cornell Medicine Institutional Animal Care and Use Committee. Neurons were derived from Sprague-Dawley rats (Charles River Laboratories strain code: 001, RRID: RGD_734476) of either sex on postnatal days 0 to 1.

### Plasmids.

The following previously published DNA constructs were used: syphy-pH (Addgene, plasmid # 220506, RRID: Addgene_220506). For this study, these new plasmids were generated: PYGB-KD (referred here as GP-KD, targeting sequence CCTGTATCCCAATGACAATTT, Addgene, plasmid # 242946, RRID: Addgene_242946), THP Synaptophysin-pH (Addgene, plasmid # 242903, RRID: Addgene_242903) and THP cyto-iATPSnFR2- miRFP670nano3 (Addgene, plasmid # 242904, RRID: Addgene_242904). All plasmids were transfected.

### Primary Neuronal Culture.

Ventral midbrain primary neuronal mixed culture (SNc and VTA) and transfections were applied as described (41). Hippocampal primary neuronal culture and transfections were applied as described (42). Energy substrates at each culture media were 1) Hippocampal culture: ~29 mM Glucose, 0.1 mg/mL transferrin, 1% Glutamax, 24 μg/mL insulin, 5% FBS, 2% N-21, 4 *μ*M ARA-C in MEM. Glucose concentration was calculated from the concentration of glucose directly added (~27.75 mM) and glucose in MEM (~5.55 mM) and FBS (~3.16 mM, might variate between batches), and weighted to their respective volumes. 2) Midbrain culture: ~17 mM Glucose, 1% Glutamax, 2% N-21, 10% FBS. Glucose concentration was calculated from the concentration of glucose in NeuroBasal (25 mM), BME (5.5 mM), and FBS (~3.16 mM, might variate between batches) and weighted to their respective volumes.

### Live-Cell Imaging.

Live-cell imaging was performed as described (43) using DIV14-18 cultured neurons. Neurons were continuously perfused at 0.1 mL/min with a Tyrode’s solution 119 mM NaCl, 2.5 mM KCl, 5 mM glucose, 50 mM HEPES, 2 mM CaCl2, 2 mM MgCl2, 50 μM DL-2-amino-5 phospho-novaleric acid (AP5), and 10 μM 6-cyano-7-nitroquinoxaline-2,3-dione (CNQX), adjusted to pH 7.4. For free glucose experiments, Tyrode’s solution osmolarity was adjusted with 55 mM HEPES. For sulpiride experiments, cultured dishes were incubated between 12 and 16 h with final drug concentration of 1 μM. When GPI was used, it was added at 10 μM final concentration in free glucose media and incubated for 5 min before electrically stimulating the neurons.

### Axonal ATP Image Analysis.

iATPSnFR2 image processing and analysis were performed as described (44). Time series of imaging pairs (miRFP670nano3 and iATPSnFR2) were split into two independent image series using a custom-written Fiji routine to facilitate analysis. ATP signals are reported as a ratio between iATPSnFR2: miRFP670nano3. Images were analyzed using the ImageJ plug-in Time Series Analyzer V3 where 40 to 60 circular ROIs of radius 1 μm corresponding to axonal terminals expressing the iATPSnFR2 were selected in the miRFP670nano3 channels, blind to the iATPSnFR2 channel, and then background subtracted. Image loading and posterior raw data saving were automatized using a homemade Python code for Fiji. Average ROIs signals were analyzed using homemade script routines in Igor-pro v6.3.7.2 (WaveMetrics, Lake Oswego, OR). ATP ratio signal was calculated by averaging all individual ROIs per neuron and normalized to the baseline.

### pHluorin Measurements.

pHluorin image processing and analysis was performed as described (45). For pHluorin experiments, nerve terminals responding to the first AP train in 5 mM glucose were selected and background subtracted. In case of the repeated stimulation assay, the traces are reported as percentage of total sensor expression by subtracting the initial fluorescence signal before stimulation and normalized to total sensor fluorescence, revealed by the 50 mM NH_4_Cl Tyrode’s solution. For single-train experiments, the traces are normalized to the fluorescence peak during the AP train.

### Immunostaining.

Immunofluorescence staining was performed as described (46). Neurons were fixed with 4% paraformaldehyde, quench PFA with 50 mM NH_4_Cl solution, permeabilized with 0.25% Triton X-100, and blocked for 10 min at room temperature with 5% BSA. Primary antibodies were diluted with 5% BSA and incubated with the cells at room temperature for 1 h. After 3× 5-min washes in phosphate-buffered saline (PBS), cells were incubated with secondary antibodies, followed by additional 3× 5-min washes in PBS. Immunofluorescence images were acquired in a similar way as live-cell imaging.

### Image Analysis and Statistics.

Live-cell images were analyzed using the ImageJ plug-in Time Series Analyzer V3 (RRID:SCR_014269), where 20 to 30 circular regions of interest (ROIs) of radius ~1 μm corresponding to synaptic boutons expressing the pHluorin (as determined with NH_4_Cl perfusion) or iATPSnFR2 (with miRFP670nano3 positive) were selected, and the fluorescence was measured over time. For Immunostained image analysis, the ROIs were selected and quantify as follows. Glycogen vs. GS experiments: Neuronal fluorescence of anti-Glycogen and anti-GS was quantified by placing ROIs around cell bodies, using the GS channel as a reference. Fluorescence intensity in each channel was normalized to the mean fluorescence of the corresponding culture (2 to 3 coverslips from a single culture batch were fixed and stained in parallel). TH-positive vs. -negative glycogen experiments: ROIs were drawn around cell bodies using the anti-TH channel as a reference. TH negative cell bodies were identified based on neuronal morphology visible in the background signal associated with the TH antibody. Glycogen mean fluorescence from TH-positive and -negative ROIs was calculated per coverslip. Sulpiride treatment experiments: ROIs were placed on axonal terminals or cell bodies using the anti-TH channel as a blinded reference. Glycogen fluorescence in sulpiride-treated samples was normalized to control (untreated) coverslips from the same culture batch and processed in parallel. Image loading and posterior raw data saving were automatized using a homemade Python code for Fiji (RRID: SCR_002285). ROIs signals were analyzed using homemade script routines in Igor-pro (Wavemetric, RRID: SCR_000325). Results of group data analysis are presented as mean ± SEM. When analyzing means, *P* values are based on the nonparametric Wilcoxon–Mann–Whitney test. *P* < 0.05 was considered significant and denoted with a single asterisk, whereas *P* < 0.01, *P* < 0.001, and *P* < 0.0001 are denoted with two, three, and four asterisks, respectively. The *n* value, indicated in the figure legends for each experiment represents the number of cells imaged, otherwise indicated.

## Supplementary Material

Appendix 01 (PDF)

## Data Availability

The data, code, protocols, and key lab materials used and generated in this study are listed in a Key Resource Table alongside their persistent identifiers at https://zenodo.org/record/16877070 (47). All data cleaning, preprocessing, analysis, and visualization was performed using Fiji (RRID: SCR_002285, (48)) and IGOR-Pro (WaveMetrics, RRID: SCR_000325). All other data are included in the manuscript and/or *SI Appendix*.
