# Supplementary material for: Neuromodulatory control of energy reserves in dopaminergic neurons
Source: Proc Natl Acad Sci U S A. 2025 Dec 5;122(50):e2523019122. doi: 10.1073/pnas.2523019122 (PMC12718339; doi:10.1073/pnas.2523019122)
Supplement: Supplementary file 1 — Appendix 01 (PDF) [file pnas.2523019122.sapp.pdf]

Supporting Information for
Neuromodulator control of energy reserves in dopaminergic neurons.

Camila Pulido[1,3], Matthew S. Gentry[2], and Timothy A. Ryan[1,3*]

[1]Department of Biochemistry, Weill Cornell Medicine, New York, NY
[2]Department of Biochemistry and Molecular Biology, University of Florida, Gainesville, FL
[3] Aligning Science Across Parkinson's (ASAP) Collaborative Research Network, Chevy Chase, MD, USA

* corresponding author **Email:** taryan@med.cornell.edu

This PDF file includes:

Figures S1 to S4

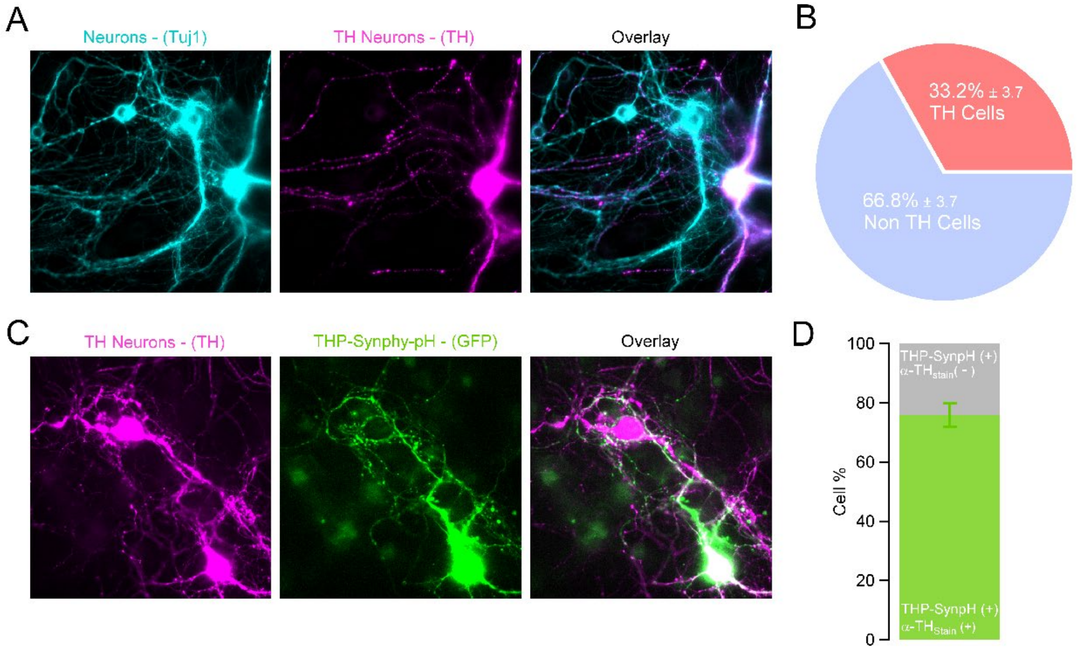

Fig. S1. Dopaminergic neurons comprise about one-third of the ventral midbrain culture and can be selectively targeted via the TH promoter. (A) Representative image of cultured neurons (anti-Tuj1; pseudo-colored cyan) that are identified as dopaminergic (TH+) neurons (anti-TH; pseudo-colored magenta). Overlay in right panel. (B) Approximately, one-third of cultured neurons are dopaminergic (n = 11 dishes). (C) Representative field of view showing that one of the dopaminergic neurons (anti-TH; pseudo-colored magenta) is transfected with Syphy-pH under TH promoter (anti-GPF; pseudo-colored green, publication). Overlay in right panel. (D) TH 75.8 ± 4% of neurons transfected with the TH promotor subsequently were identified as dopaminergic (n = 21 dishes).

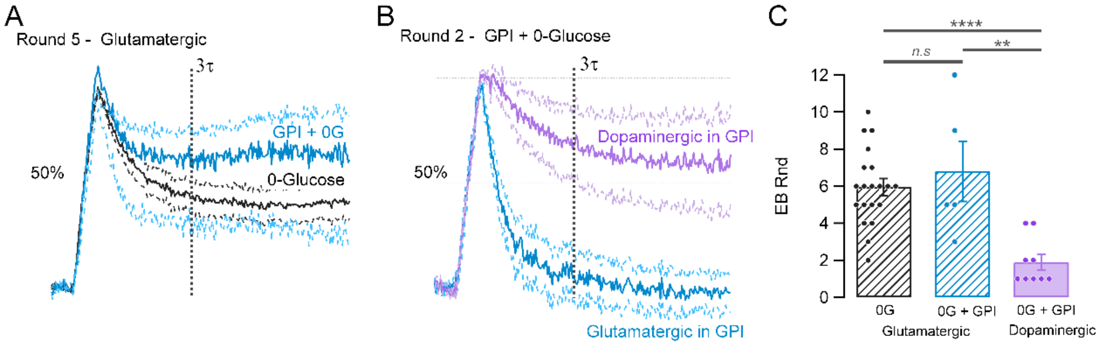

Fig. S2. Glutamatergic neurons do not rely on glycogen under hypoglycemic conditions. (A) Average traces at the fifth round of stimulation (50AP, 10Hz) in absence of glucose in control glutamatergic neurons (gray trace; n = 20) versus glutamatergic neurons treated with GPI (blue trace; n = 5) shows that both populations are equally functionally impaired. (B) Second round of stimulation comparison between dopaminergic versus glutamatergic neurons treated with GPI shows that early on dopaminergic SV recycling is affected due to lack of access to glycogen, whereas glutamatergic neurons are not affected by GPI treatment. (A-B) Responses are normalized to the peak. Vertical dashed line represents three times t measured at 5mM glucose response. Horizontal dashed line represents 50% retrieval of the exocytic signal. (C) Number of rounds of stimulation before EB exceeds 50% is equally higher in glutamatergic neurons with or without GPI than in Dopaminergic neurons in GPI.

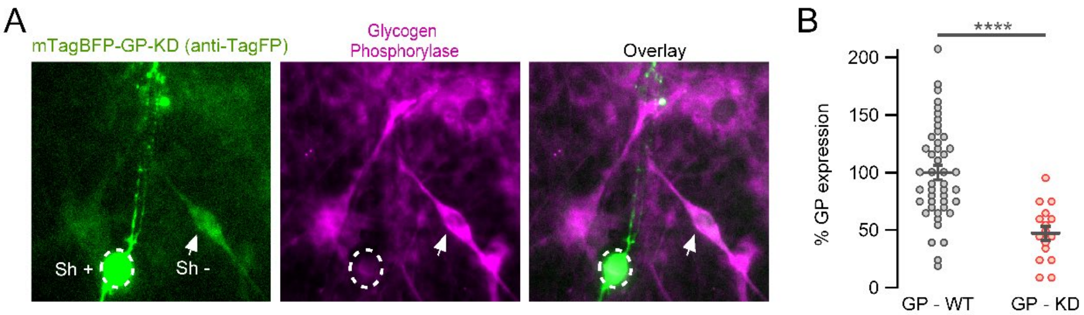

Fig. S3. Expression of a ShRNA targeting Glycogen phosphorylase resulted in ~35% suppression of GP expression. (A) Representative image from a neuron expressing an mTagBFP-GP-Sh (stained with FlutoTag-X2 anti-TagFP Atto488). BFP-positive cell body (dashed white circle) show much lower GP immunofluorescence when compared with neighboring non-transfected cell body (white arrow). (B) Average expression of GP in cell bodies transfected with mTagBFP-GP-Sh (n = 16) normalized to their respective neighboring non-transfected cell bodies (n = 45) from 3 cultures. Respectively as: mean ± SEM: 47.25%± 6.0 vs 100% ± 6.2. ****P<0.0001, Wilcoxon-Mann-Whitney test.

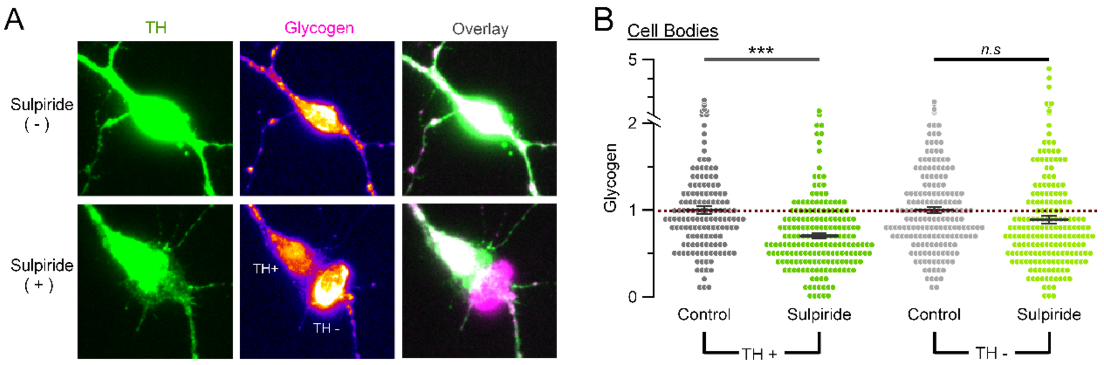

Fig. S4. Sulpiride decrease glycogen levels exclusively in dopamine neurons, which are modulated by D2 auto-receptors. (A) Representative images of cell bodies from dopamine neurons (anti-TH, green) with (bottom) or without (top) overnight 1uM sulpiride treatment; and their respective glycogen level content (color-code from dark violet = low levels to yellow = high levels; same scale top and bottom). The glycogen field of view image treated with sulpiride shows a comparison between a TH+ versus a non-TH neuron. (B) Average glycogen expression from cell bodies normalized to control of TH+ (dark gray dots; n = 146) and to control of TH-populations (light gray dots; n = 183), shows that glycogen storage levels decrease by a 29.76% ± 2.94 only in neurons that are dopaminergic (TH+) and that were treated with sulpiride (dark green dots; n = 193 from same cultures than TH+ control). ***P<0.001, non-significant as ns, Wilcoxon-Mann-Whitney test.
